# Treatment of COPD: the simplicity is a resolved complexity

**DOI:** 10.1186/s40248-019-0181-8

**Published:** 2019-06-03

**Authors:** Marco Candela, Rosario Costorella, Annalisa Stassaldi, Vanessa Maestrini, Giacomo Curradi

**Affiliations:** 1 Director of the Department of Medicina Area Vasta 2, ASUR Marche, Via Aldo Moro 25, Jesi (AN), 60035 Italy; 2 Medical & Scientific Department, GlaxoSmithKline Spa - Pharmaceutical, Via Fleming 2, Verona, 37135 Italy

## Background

The introduction of simple and reproducible functional parameters as Forced Expiratory Volume in 1 s (FEV_1_) and Forced Vital Capacity (FVC) has been fundamental to diagnose, classify and measure the progression of Chronic Obstructive Pulmonary Disease (COPD).

However, it has been recently emphasized that these parameters do not allow an overview of the complexity and heterogeneity of COPD. In fact, the disease can show several intrapulmonary and extrapulmonary components with non-linear dynamic interactions of which not all these components are present in all patients nor in individual patients, at all time points [1].

These differences have justified the recognition of different phenotypes among patients with COPD. The objective of this review is to overhaul the evidences recently published in order to define COPD characteristics able to suggest a therapeutic algorithm.

### COPD phenotypes

The term “phenotype” was used for the first time by Wilhelm Johannsen in 1909, together with the term “genotype”, in order to describe two different levels of realities that are closely linked [2]. Since, the concept of phenotype has been taken in consideration by different specialties in medicine to explain a specific clinical presentation of the same disease: COPD is an example. A better definition of phenotypes is important not only for an improved understanding of the underlying disease processes, but also for the clinical and therapeutic implications.

The acronym “COPD” evolved to describe two distinct pathological disease processes into a single clinical entity that is mainly linked to cigarette smoking (chronic bronchitis and lung emphysema) [3].

In fact, based on clinical, pathological and radiological features, two main phenotypes have been identified: type A patient or “pink puffer” (emphysema) and type B patient or blue-bloater (chronic bronchitis) [4].

In type A patients the dominant symptom is dyspnea, while cough and hypersecretion are modest. Type A patients show radiological evidences of emphysema and rarely hypercapnia or recurrent heart failure, instead lung volumes are generally increased and diffusing capacity for carbon monoxide (D_L_CO) is impaired, mainly due to a not homogenous ventilation and a ventilation-perfusion mismatch. Emphysema severity is independently correlated with a rapid annual decline in FEV_1_ [5].

In type B patients the main symptom is mucous hypersecretion, while dyspnea is modest. Type B patients often show hypercapnia and hypoxemia with secondary pulmonary hypertension and cardiovascular comorbidities, while lung volumes are not increased and diffusing capacity for carbon monoxide is usually preserved. The hypoxemia in the gas exchange, in turn, stimulate pulmonary vasoconstriction and increase of erythropoiesis. The oxyhemoglobin desaturation and erythrocytosis combine to produce the typical cyanosis of patient known as “blue-bloater”.

Airflow obstruction is influenced by both small airway disease and emphysema. The small conducting airways are the major site of airflow obstruction in chronic obstructive pulmonary disease, and histologic data suggest that small airway abnormality may precede emphysema [6]. Interestingly a recent study by Kirby et al. demonstrated that airway count by Computer Tomography (CT) is significantly reduced in mild COPD independently of emphysema severity and in particular parent airways with missing daughter branches had reduced inner diameters and thinner walls compared with those without missing daughter branches.

The reduction of CT airway count is significantly and independently associated with rapid decline in lung function over time. These findings indicate that early airway-related changes can be assessed in vivo using CT and suggest that early intervention may be required for optimal disease modification [7].

In the clinical setting, emphysema and bronchitis/bronchiolitis often coexist with different degree of severity in the same patient making it very difficult to physiologically and clinically identify the contribution of each. Thus, such overlap led to the terminology of COPD.

Following the evolution in COPD knowledge, Han and collaborators in 2010 proposed that COPD phenotypes should be associated with clinically meaningful outcomes. This more focused definition allows for classification of patients into distinct prognostic and therapeutic subgroups for both clinical and research purposes [8].

With this background the 2001 “Global Strategy for the Diagnosis, Management and Prevention of COPD” (GOLD) created a new COPD classification based on the severity of airflow limitation as defined by FEV_1_ values, which was widely used with minor changes until 2011. However, FEV_1_ alone is an insufficient parameter to characterize the complexity and severity of COPD and to guide its treatment. In 2011, the GOLD committee proposed a three-dimensional assessment of COPD, considering the severity of airflow limitation, the level of symptoms and the previous history of exacerbations. From the edition of 2017, confirmed also in the latest edition 2018, the GOLD document was amended and several new concepts have been introduced: the classification of airflow limitation severity in COPD, based on post-bronchodilator FEV_1_ in the presence of a reduced FEV_1_/FVC ratio (the* sine qua non *for diagnosing COPD) is no longer the sole criterion to establish the appropriate therapy for a given patient, but has still a diagnostic and prognostic value [9].

Today this approach does not seem to take in consideration the association of the two original conditions and phenotypes (type A and B), often overlapped in a wide range of degrees, but constantly evolving in new and intermediate phenotypes, allowing more than one therapeutic option.

In this context, a third phenotype of COPD, albeit debatable, is generally known as ACO (Asthma COPD Overlap). This population represents about 20% of patients with obstructive disorders of the lower respiratory tract and the main feature is a persistent airflow limitation not fully reversible but comparable to asthma, with a worse prognosis compared with patients who are suffering from asthma or COPD alone. In particular, such patients could experience a larger number and more severe episodes of exacerbations [10].

In the assessment of COPD another endpoint that may define a clinical phenotype is the exacerbation rate. COPD exacerbations have a great impact on health, both short and long term, and negatively influence the natural history and rate of disease progression [11].

The ECLIPSE study also showed that a significant proportion of patients with mild or moderate disease experienced frequent exacerbations regardless of the severity of the airflow obstruction [12]. This patients’ subset was called “COPD phenotype with frequent exacerbations”.

Finally, in the non-proportional Venn diagram of COPD by March et al., ten different subsets of disease could be recognized. The diagram shows the huge heterogeneity of this disease and that could be the reason why some patients could have a late diagnosis [13].

### A possible complexity resolution

A critical question is whether it is useful to proffer different phenotypes of COPD if standard criteria or definitions do not exist to clearly identify each of them. One objective of future clinical research in COPD should be to determine whether new and more precise phenotypes, as well as to provide clear definitions surrounding the various phenotypes identified perhaps by identifying clusters of constant phenotypes, would better guide clinical evaluation and therapeutic strategies.

How can this be done?

At present, it is possible to identify clinical, radiological or physiological features that might facilitate determining whether a patient has “predominant” emphysema or “predominant” chronic bronchitis-like phenotype. Izquierdo-Alonso and colleagues determined through clinical and radiographic tests (such as diffusion test, CT and thorax radiography) the prevalence of phenotypes in COPD patients showing that 43.2% had emphysematous phenotype, 44.7% had chronic bronchitis and the other 12.1% showed mixed characteristics with asthma [14]. “Emphysema” patients showed significantly lower FEV_1_ values in comparison with other and greater levels of dyspnea (*p* < 0.05), although there were no differences in the use of hospital health care resources. “Chronic bronchitis” patients showed a greater prevalence of cardiovascular comorbidities and of sleep apnea syndrome.

According to the results of COPD Gene study [15], it was possible to characterize phenotype with high-resolution computed tomography (HRCT) using the percentage of emphysema (with a − 950-Hounsfield units threshold) and the level of bronchial wall thickness: in the emphysema predominant group the percentage of emphysema was ≥35% and the bronchial wall thickness < 1.75 mm; while in the small airway disease predominant group, the level of emphysema was < 35% with a bronchial wall thickness ≥ 1.75 mm.

These differential criteria correlated with interesting clinical and functional parameters, for example worse dyspnea, lower level of FEV_1_, significantly higher body indexes and osteoporosis were more frequent in the emphysema predominant group. Conversely, the increase in wall thickness detected with the HRCT was associated with greater exacerbation frequency in the small airway disease predominant group.

Using clinical (i.e.sputum characteristics), pulmonary function data (FEV_1_/FVC, TLC, FRC and D_L_CO), along with computed tomography assessment of lung density, one can propose clinical COPD phenotypes using multivariate models [16]. Finally, an indication about the response to combined inhaled long acting beta-2-agonist and inhaled corticosteroid therapy among different COPD subtypes has been shown in the clinical trial of Lee and collaborators. Despite the small number of patients, the response varies significantly among different COPD subtypes with no improvement in FEV_1_ or dyspnea after the 3-month treatment period in emphysema-dominant patients [17].

### Current COPD treatment choices based on symptomatic & exacerbation phenotypes

Regarding the different COPD phenotypes, a question remains as to which pharmacologic agent (s), i.e. β_2_ agonists, antimuscarinics, inhaled or systemic corticosteroids, theophylline, phosphodiesterase-4 (PDE4) inhibitors, mucolytics and macrolides would be optimal for a given phenotype.

As previously shown by Anthonisen et al., special programs in supporting smoking cessation can achieve a reduction in terms of all-cause mortality (8.83 per 1000 person-years vs. 10.38 per 1000 person-years; p 0.03), even if those interventions are successful only in a minority of patients [18]. One explanation of a better survival in former smokers is partly attributable to the prevention of smoking damage over time (lower functional decay of the lung [19] and increased risk of cancer and cardiovascular diseases in smoker [18]) and partly to the greater pharmacological efficacy of compounds containing ICS [20].

Interestingly, in a recent Delphi consensus project run in Italy, the most effective step to reduce lung functional decline were considered by the 207 specialists interviewed to be smoking cessation [21].

Therefore, smoking cessation support programs should always be provided in the patient with COPD.

**Fig. 1:**
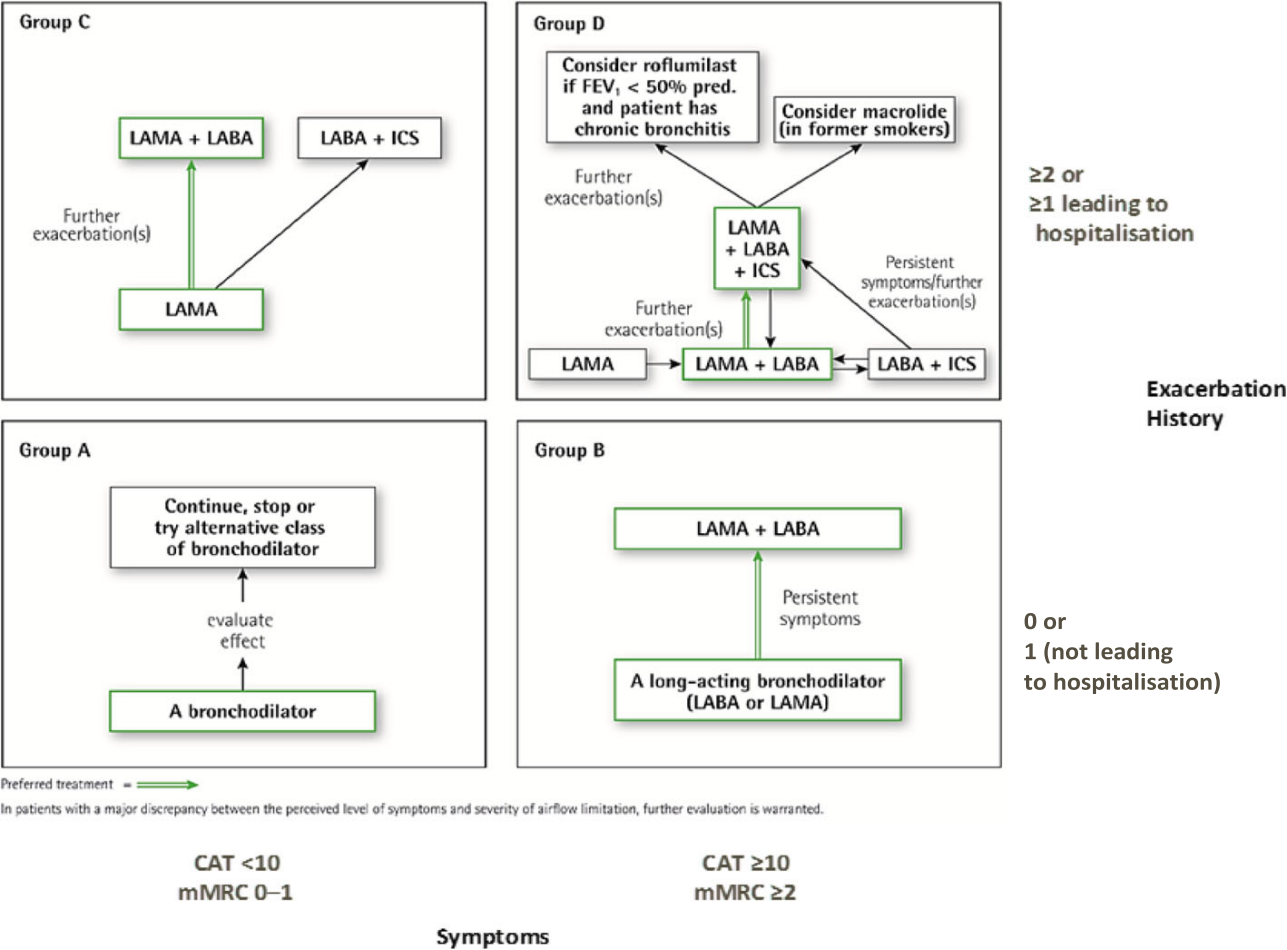
Pharmacologic treatment algorithms by GOLD grade

The 2018 GOLD strategy document proposes treatment recommendations based on COPD phenotypes defined by symptoms and exacerbation history as outlined in Fig. 1 with preferred treatments highlighted in green.

Furthermore, GOLD also states potential consideration should be given to step down from triple therapy to a non-ICS treatment in case of ascertained pneumonia risk (e.g. on an ICS-containing regimen) or lack of marked clinical improvement. Bronchodilators, including dual bronchodilators, figure prominently into the treatment guidelines. This is most likely due to the increasing amount of evidence supporting the benefits LAMA/LABA combinations on lung and symptom improvement with no increase in side effects compared to LAMA or LABA alone [22]. Recent findings from the FLAME study also demonstrated an exacerbation benefit with LAMA/LABA vs ICS/LABA. On the other hand, combination ICS/LABA have also shown benefit in reducing exacerbations and improving lung function and health-related quality of life compared to placebo, ICS and LABA alone [23–25]. However, ICS-containing therapy is associated with an increased risk of pneumonia with no association with an increase in mortality supporting a favorable benefit/risk profile in patients at risk of exacerbations [26–28].

As with all therapeutic choices, an assessment of benefit/risk should be made on an individual patient level and those with COPD receiving inhaled corticosteroids should be carefully evaluated to identify concomitant osteoporosis and diabetes, and monitored for progression of these diseases for early implementation of appropriate therapy.

Recently the publication of the IMPACT Study (**I**nfor**M**ing the **PA**thway of **C**OPD **T**reatment) has shown new evidences about the role of single inhaler triple therapy (ICS/LABA/LAMA) compared to ICS/LABA and LAMA/LABA [29]. The main results of this study were obtained on: reduction of exacerbation rate, lung function improvement (in terms of trough FEV_1_ improvement), mortality data and incidence of pneumonia. Single inhaler triple therapy with FF/UMEC/VI demonstrated a significant reduction of moderate/severe exacerbation rate and prolonging the time to first exacerbation on-treatment compared to both FF/VI and UMEC/VI. A reduction in the number hospitalizations was seen with FF/UMEC/VI vs UMEC/VI but not FF/VI. There was a significant improvement in lung function with FF/UMEC/VI compared with both dual treatments (ICS/LABA and LAMA/LABA); this result reinforces the need for maximum bronchodilation to optimize lung function improvements.

Some debate was recently made on the asthmatic patient enrolled in the IMPACT study: only patients with COPD diagnosis, based on ATS/ERS criteria were included and patients were permitted to enter the study if they also had a prior history of asthma, without an ongoing diagnosis of asthma, associated to other parameters as 65 years of age, substantial smoking history and a high frequency of exacerbations observed during the 52-week treatment period (~ 1 event/patient/year) [29]. Furthermore, of the population enrolled in IMPACT, 18% had airflow limitation that was reversible to salbutamol and this proportion is lower than that shown in other studies in COPD population [30–32].

Taken together, these characteristics should be obviously ascribed to COPD population rather than to an asthma population. For over a decade the respiratory community has debated the potential mortality benefits of ICS-containing treatments in COPD, but until now this benefit has not been prospectively shown [33–35].

FF/UMEC/VI and FF/VI are the only COPD medications available that have prospective data showing a reduction in the risk of all-cause mortality vs LAMA/LABA (UMEC/VI).

There was an increase in the risk of pneumonia seen with FF/UMEC/VI and FF/VI vs UMEC/VI which was expected and consistent with the class of ICS containing therapy. As indicated previously pneumonia and exacerbations are key events during COPD disease, with different implications for individual patients. It is important to consider both events, as viewing them in tandem may provide a better picture of the overall benefit/risk profile of a particular COPD therapy.

The IMPACT trial is the only clinical study which has directly compared all three major inhaled therapy combinations available (ICS/LABA, LAMA/LABA and ICS/LAMA/LABA) in the treatment of COPD. The direct comparison between these therapies has helped to better understand the role of ICS on top of maximal bronchodilation with LAMA/LABA.

Macrolides have demonstrated a measurable efficacy in preventing exacerbations. However, their use in a chronic/preventive manner needs to be decided carefully balancing the potential efficacy in the right patients with the potential risk connected to an antibiotic overuse and potential antibiotic resistance in a single patient and/or a community [36].

Roflumilast, the first phosphodiesterase-4 inhibitor available, is indicated for maintenance treatment of severe COPD (FEV_1_ post-bronchodilator less than 50% predicted) associated with chronic bronchitis in adult patients with a history of frequent exacerbations as add on to bronchodilator treatment [37]. In fact, a *post-hoc *pooled analysis by Rennard et al., showed that roflumilast reduced exacerbation frequency manly in a subset of COPD patients whose characteristics included chronic bronchitis with/without concurrent ICS [38].

In terms of oxygen supplementation, it’s well known and accepted that in patients with chronic obstructive pulmonary disease (COPD) and chronic hypoxemia long-term oxygen administration can improve pulmonary hypertension and increase exercise performance [39]. However only two studies have shown in the early 1980s that the use of long term oxygen therapy (LTOT) can lower mortality in patient with COPD associated to chronic hypoxemia. In the Medical Research Council trial, 87 COPD patients were randomized to an LTOT group that received oxygen for at least 15 h per day or to a no-oxygen control group. Within the 5-year study period, 19 out of 42 died in the treated group versus 30 out of 45 in the control group (probability of survival was 55% versus 33% respectively with a *p* < 0.05) [40].

The Nocturnal Oxygen Therapy Trial (NOTT) compared continuous (24-h) oxygen administration with 12-h nocturnal oxygen supplementation over a period of two years; patients treated with 24 oxygen showed a significant improvement in survival versus those given 12-h nocturnal oxygen (mean annual death rate was 11,9% and 20,6% respectively with a p < 0.05) [41].

Currently the British Thoracic Society (BTS) guidelines suggest using LTOT in patients with stable chronic obstructive pulmonary disease (COPD) and a resting PaO_2_ ≤ 55 mmHg and in patients with stable COPD with a resting PaO_2_ ≤ 60 kPa associated with evidence of peripheral edema, polycythemia (hematocrit ≥55%) or pulmonary hypertension independently if patient is a prevalent bronchitis or emphysema [42].

As recently shown in an interesting review by Minervini et al., a quite limited and well selected group of COPD patients can benefit from surgical and endoscopic lung volume reduction (LVRS and ELVR, respectively). These treatments should be considered in presence of heterogeneous emphysema (upper lobe predominant), severe obstruction (FEV_1_ ≤ 45% but > 20% predicted), limited exercise capacity with hyperinflated lung and moderate impairment of the lung diffusion capacity (at least D_L_CO > 20% predicted) [43]. However, to date, there are no data comparing the two techniques and other studies should be conducted in order to clarify long term outcomes, side effect and costs linked to these different approaches.

A non-pharmacological treatment for COPD patients is represented by the pulmonary rehabilitation. The topic is still debated and controversial especially in COPD patients after a recent exacerbation due to conflicting evidences emerged from more recent trials: these last showed no benefit of rehabilitation on hospital readmissions and mortality versus older studies [44]. Nevertheless, the BTS guidelines suggest the use of respiratory rehabilitation in COPD patients having a view to improving: exercise capacity, dyspnea, health status and psychological wellbeing [45]. To date, the advantages of respiratory rehabilitation do not seem to be associated with the prevalent bronchitis or emphysematous status.

## Conclusion

Considering the treatment recommendations by GOLD based on the symptomatic and/or exacerbation phenotypes, are there other phenotypes that can be identified to tailor the right therapy to the right patient?

For example, an approach to COPD pharmacotherapy used by Spanish investigators utilizes an easy table of four major phenotypes (Non exacerbators, ACO, exacerbators with emphysema, exacerbators without emphysema) with five treatments options (bronchodilators, ICS, mucolytics, PDE4 inhibitors, macrolides), linking each of the four phenotypes with the appropriate treatment(s) [46]. Furthermore, a recent paper emphasizes the notion of phenotyping COPD patients before starting treatment, by recommending that inflammatory phenotypes, such as chronic bronchitis, frequent exacerbators and those with multiple co-morbidities need ICS therapy; and patients that are emphysematous with dyspnea and lung hyperinflation, fast decliners, need dual bronchodilation with LABA/LAMA [47].

**Fig. 2:**
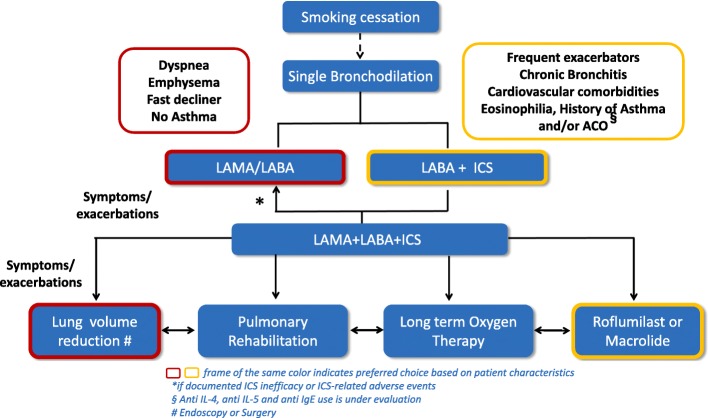
Treatment Algorithm for COPD

Based on these considerations we propose a treatment algorithm easily summarized in Fig. 2, useful both in the phase of discharge and outpatient visit. This pathway will need to be confirmed through additional clinical trials and evidence, that will also consider a validated set of phenotyping criteria.

Our aim is to stimulate a common thought: to consider again, in a revised form, “chronic bronchitis” and “emphysema” in terms of impact on each patient’s health and life, in order to personalize as much as possible assessment and treatment.

In other words, should we prefer a modern redefinition of two old concepts rather than, with absolute gratitude, the ‘old’ term COPD? The latest scientific evidence is helping to identify and possibly clarify these different patient phenotypes which could be crucial in determining the right treatment for the right patient.

## Abbreviations

ACO: Asthma COPD Overlap
ATS/ERS: American Thoracic Society/European Respiratory Society
BTS: British Thoracic Society
CAT: COPD assessment test
COPD: Chronic Obstructive Pulmonary Disease
CT: computer tomography
D_L_CO: Diffusing capacity for carbon monoxide
ELVR: Endoscopic Lung Volume Reduction
FEV_1_: Forced Expiratory Volume in 1 s
FF: Fluticasone Furoate
FRC: Functional residual capacity
FVC: Forced Vital Capacity
GOLD: Global Initiative for chronic Obstructive Lung Disease
HRTC: High-resolution computed tomography
ICS: Inhaled corticosteroid
IMPACT: InforMing the PAthway of COPD Treatment
LABA: Long acting beta 2 agonist
LAMA: Long acting anti-muscarinic agent
LTOT: Long term oxygen treatment
LVRS: Lung Volume Reduction Surgery
MMRC dyspnea scale: Modified Medical Respiratory Council dyspnea scale
NOTT: Nocturnal Oxygen Therapy Trial
PDE4: Phosphodiesterase-4 inhibitor
TLC: Total lung capacity
UMEC: Umeclidinium
VI: Vilanterol
